# Supplementary material for: Splice-Junction-Based Mapping of Alternative Isoforms in the Human Proteome
Source: Cell Rep. Author manuscript; Available in PMC 2020 Jan 15. (PMC6961840; doi:10.1016/j.celrep.2019.11.026)
Supplement: 2 [file NIHMS1546469-supplement-2.zip › DF1/PXD006675/PulmonaryVein/PulmonaryVein_12_TTN_RVEAEPAEVTEFR.pdf]

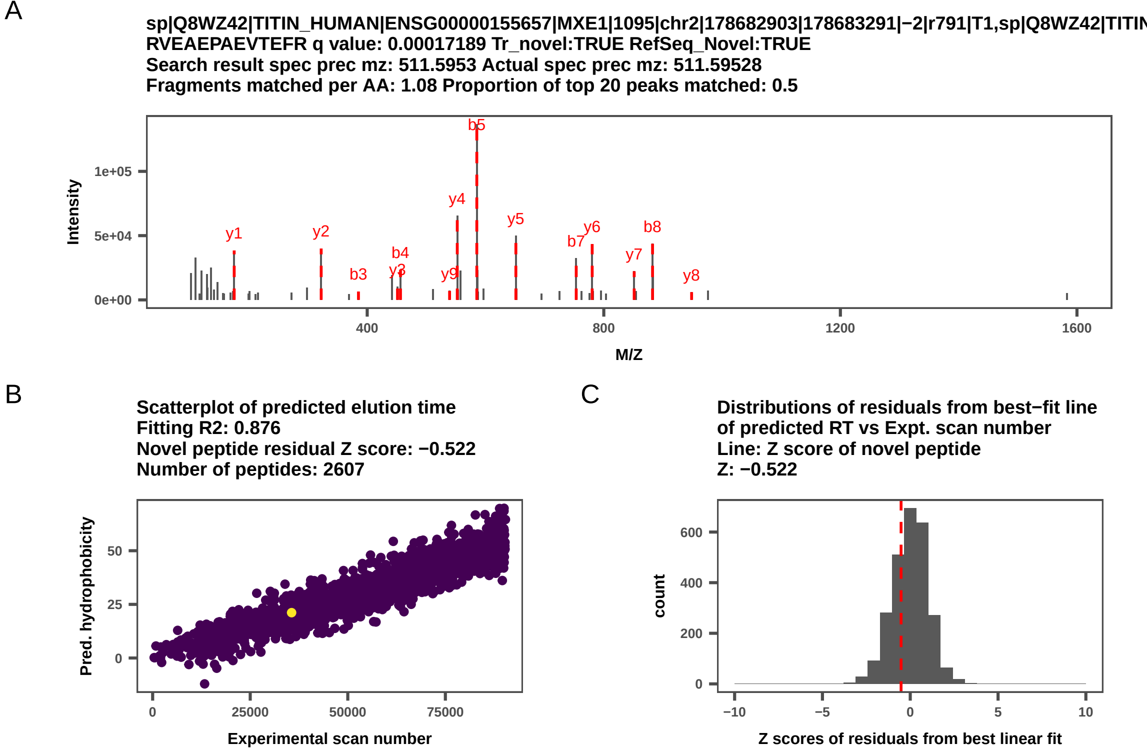
A
sp|Q8WZ42|TITIN_HUMAN|ENSG00000155657|MXE1|1095|chr2|178682903|178683291|−2|r791|T1,sp|Q8WZ42|TITIN
RVEAEPAEVTEFR q value: 0.00017189 Tr_novel:TRUE RefSeq_Novel:TRUE
Search result spec prec mz: 511.5953 Actual spec prec mz: 511.59528
Fragments matched per AA: 1.08 Proportion of top 20 peaks matched: 0.5
Intensity
M/Z
b5
y4
y5
y6
b8
b7
y1
y2
b4
y7
b3
y3
y9
y8
B
Scatterplot of predicted elution time
Fitting R2: 0.876
Novel peptide residual Z score: −0.522
Number of peptides: 2607
Pred. hydrophobicity
Experimental scan number
C
Distributions of residuals from best−fit line
of predicted RT vs Expt. scan number
Line: Z score of novel peptide
Z: −0.522
count
Z scores of residuals from best linear fit
